# Prognostic impact of GNRI-IPI score in octogenarians with diffuse large B-Cell lymphoma treated with R-CHOP

**DOI:** 10.1007/s00277-025-06670-x

**Published:** 2025-10-14

**Authors:** Eun-Jeong Jeong, Woochan Park, Jeongmin Seo, Minsu Kang, Eun Hee Jung, Sang-A Kim, Koung Jin Suh, Ji-Won Kim, Se Hyun Kim, Jeong-Ok Lee, Jin Won Kim, Yu Jung Kim, Keun-Wook Lee, Jee Hyun Kim, Jong Seok Lee, Soo-Mee Bang, Ji Yun Lee

**Affiliations:** Department of Internal Medicine, Seoul National University College of Medicine, Seoul National University Bundang Hospital, Seongnam, Republic of Korea

**Keywords:** Diffuse large b-cell lymphoma, R-CHOP, Elderly, Survival, Prognostic factors, Nutritional status

## Abstract

Diffuse large B-cell lymphoma (DLBCL) outcomes decline in patients aged ≥ 80 years, with R-CHOP as standard therapy despite frequent dose reductions due to frailty. This study evaluates treatment outcomes and prognostic factors, including the Geriatric Nutritional Risk Index (GNRI), in this population. We retrospectively analyzed 102 patients aged ≥ 80 years with newly diagnosed DLBCL treated with full-dose or dose-attenuated R-CHOP at Seoul National University Bundang Hospital (2005–2024). Baseline characteristics, treatment delivery, toxicities, and survival were assessed using Kaplan-Meier methods and Cox proportional hazards models. Median age was 83 years; 57.8% had advanced-stage disease, and 55.8% were high/high-intermediate IPI risk. Median relative dose intensity was 54.0%; 65.7% completed treatment. Grade 3–4 toxicities occurred in 33.3%. At 2 years, overall survival (OS) was 71.8% and progression-free survival (PFS) was 67.2%. In multivariate analysis, low GNRI (< 81.6) independently predicted worse OS (HR 3.09, 95% CI 1.40–6.82, *p* = 0.005) and PFS (HR 2.84, 95% CI 1.34–6.02, *p* = 0.006); IPI ≥ 3 was associated with inferior PFS (HR 2.11, 95% CI 1.03–4.32, *p* = 0.042). The composite GNRI-IPI score stratified OS and PFS effectively, with high-risk showing worse survival (OS: HR 6.44, 95% CI 2.45–16.93, *p* < 0.001; PFS: HR 6.00, 95% CI 2.42–14.92, *p* < 0.001) and higher grade 3–4 toxicities (63.6% vs. 22.0% in low-risk, *p* = 0.023). GNRI-IPI enhances risk stratification and toxicity prediction in very elderly DLBCL patients, supporting nutritional integration in prognostication. Prospective validation is warranted.

**Supplementary Information:**

The online version contains supplementary material available at 10.1007/s00277-025-06670-x.

## Introduction

Diffuse large B-cell lymphoma (DLBCL) is the most common subtype of non-Hodgkin lymphoma, accounting for approximately 30–40% of cases worldwide [1, 2]. The introduction of CD20-targeted monoclonal antibody therapy, particularly rituximab, has significantly improved treatment outcomes, establishing R-CHOP (rituximab, cyclophosphamide, doxorubicin, vincristine, and prednisolone) as the standard first-line regimen [3]. Large, randomized phase III trials have demonstrated substantial survival benefits, with 5-year overall survival (OS) rates exceeding 70% in patients younger than 70 years [3–5]. However, survival outcomes decline markedly with increasing age [6]. For instance, studies report 5-year OS of 40–50% in patients over 70 years, decreasing to approximately 39% at 2 years for those aged ≥ 80 years [7–9]. For this population, the National Comprehensive Cancer Network (NCCN) guidelines recommend R-miniCHOP—a dose-attenuated version of R-CHOP with approximately 50% dose reduction—as the standard of care, based on phase II study results that report 2-year overall survival (OS) rates of 59% and 2-year progression-free survival (PFS) rates of 47% [10, 11]. In real-world clinical practice, many elderly patients struggle to tolerate even dose-attenuated R-CHOP due to age-related comorbidities, functional decline, and organ dysfunction, often necessitating further dose reductions or low-intensity regimens [12]. These modifications, driven by concerns over treatment-related toxicity, may compromise therapeutic efficacy and contribute to suboptimal outcomes [13].

Prognostic evaluation in DLBCL is typically performed using the International Prognostic Index (IPI), which incorporates age, disease stage, serum lactate dehydrogenase (LDH) level, Eastern Cooperative Oncology Group (ECOG) performance status, and number of extranodal sites [14]. However, the IPI may not fully capture the heterogeneity and unique clinical challenges faced by patients aged 80 years or older, potentially limiting its discriminatory power in this age group [15, 16]. Nutritional status, assessed by the Geriatric Nutritional Risk Index (GNRI), reflects malnutrition and frailty, which are prevalent in elderly patients and associated with poorer outcomes in hematologic malignancies [17, 18]. Given the increasing number of very elderly DLBCL patients and their declining survival rates with age, we aimed to evaluate treatment outcomes and identify prognostic factors, including the role of GNRI, to optimize R-CHOP-based therapy in this population.

## Methods

### Study design and patient selection

This was a single-center, retrospective study conducted at Seoul National University Bundang Hospital, a tertiary care hematology-oncology center. We reviewed the medical records of patients with newly diagnosed DLBCL, aged 80 years or older, who received at least one cycle of full-dose or dose-attenuated R-CHOP chemotherapy as their initial treatment from March 2005 to December 2024. All cases were histologically confirmed as DLBCL according to the World Health Organization (WHO) classification and were further classified into germinal center B-cell-like (GCB) and non-GCB subtypes using the Hans algorithm with immunohistochemical (IHC) markers (CD10, BCL6, and MUM1). Patients were excluded if they had the following: (1) a previous history of lymphoma, (2) transformed lymphoma, (3) primary central nervous system DLBCL, (4) primary mediastinal B-cell lymphoma, or (5) had received initial chemotherapy regimens other than R-CHOP. A total of 102 patients aged 80 years or older at diagnosis were included in the analysis. Because this was a single-center retrospective cohort, we consecutively enrolled all eligible patients without a priori sample-size calculation.

The study was approved by the Institutional Review Board of Seoul National University Bundang Hospital (IRB number: B-2412-942-101), with informed consent waived due to its retrospective nature. Patient data were anonymized and handled confidentially in accordance with the Declaration of Helsinki and relevant data protection regulations.

### Treatment protocol

The standard R-CHOP regimen consisted of rituximab 375 mg/m², cyclophosphamide 750 mg/m², doxorubicin 50 mg/m², and vincristine 1.4 mg/m² (maximum dose 2 mg) administered intravenously on day 1, along with a tapering dose of oral prednisolone (40 mg on day 1, 30 mg on days 2–3, and 30 mg on days 4–5). Chemotherapy cycles were repeated every 21 days for up to 6 cycles, depending on patient tolerance and response assessment. Dose modifications were made at the discretion of the treating physician based on patients’ performance status, comorbidities, organ function, and treatment tolerability.

### Clinical assessments and definitions

Baseline clinical characteristics, including age, sex, ECOG performance status, Ann Arbor stage, serum LDH level, number of extranodal sites, and IPI score were collected from medical records. The Charlson Comorbidity Index (CCI) was calculated according to the original scoring system proposed by Charlson et al. [19]. The GNRI was calculated as: GNRI = (1.487 × serum albumin [g/L]) + (41.7 × current body weight/ideal body weight), with ideal body weight = height² (m) × 22 [17]. Relative dose intensity (RDI) was defined as the ratio of the actual delivered dose intensity to the standard planned dose intensity for each chemotherapy agent, expressed as a percentage. Total RDI was calculated as the average RDI across all chemotherapy agents administered during the entire treatment course. Treatment-related toxicities were assessed and graded using the National Cancer Institute’s Common Terminology Criteria for Adverse Events (CTCAE) version 5.0. The primary endpoint was OS, defined as the time from diagnosis to death from any cause or last follow-up. Secondary endpoints included PFS, defined as the time from diagnosis to disease progression, relapse, death from any cause, or last follow-up, treatment completion rates, and grade 3–4 toxicities.

### Statistical analysis

Categorical variables were compared using the Chi-square test or Fisher’s exact test, as appropriate. Continuous variables were analyzed using the Mann-Whitney U test or Student’s t-test, depending on the distribution of the data. To determine the optimal cut-off point for the GNRI in predicting overall survival, we applied maximally selected rank statistics using the maxstat.test function from the maxstat package in R [20]. This method identifies the GNRI threshold that best discriminates survival outcomes based on the log-rank test. The optimal cut-off was identified as 81.6 (*p* = 0.022). We conducted bootstrap validation (B = 500) of the GNRI cut-off (81.6); in each resample, we re-estimated the optimal cut-off using maximally selected rank statistics and assessed log-rank significance at 81.6 [20]. To further evaluate model performance, we calculated Net Reclassification Improvement (NRI) and Integrated Discrimination Improvement (IDI) using Cox proportional hazards models, comparing GNRI-IPI and IPI predictions at 24 months. Decision curve analysis (DCA) was performed using time-specific binary outcomes and Cox-derived risks across clinically relevant thresholds (5–30%) to assess net benefit (NB).

Survival curves were constructed using the Kaplan-Meier method, and differences between groups were compared using the log-rank test. Univariate and multivariate analyses were performed using Cox proportional hazards regression models to identify prognostic factors, with hazard ratios (HRs) and 95% confidence intervals (CIs) reported. A novel prognostic score (GNRI-IPI) was developed by combining GNRI and IPI. Patients were stratified into three risk groups: low risk (GNRI ≥ 81.6 and IPI < 3), intermediate risk (either GNRI < 81.6 or IPI ≥ 3), and high risk (GNRI < 81.6 and IPI ≥ 3). The GNRI-IPI score’s prognostic value was assessed using Cox models and Kaplan-Meier curves for OS and PFS. The predictive accuracy of the GNRI-IPI score was compared with IPI alone using Harrell’s concordance index. Associations between the GNRI-IPI score and clinical outcomes (early treatment termination, grade 3–4 toxicities) were evaluated using the Chi-square test or Fisher’s exact test, as appropriate. Statistical significance was set at *p* < 0.05. Statistical analyses were performed using R statistical software version 4.5.1 (R Foundation for Statistical Computing, Vienna, Austria).

## Results

### Baseline patient characteristics

A total of 102 patients aged 80 years or older with DLBCL were included in this analysis, with demographic and baseline disease characteristics detailed in Table 1. The median age was 83.0 years (interquartile range [IQR], 81.0–85.0), with 33 patients (32.4%) aged ≥ 85 years. The cohort included 66 males (64.7%) and 36 females (35.3%), with 38 patients (37.3%) having an ECOG performance status ≥ 2. Comorbidity burden, assessed by the CCI, showed 36 patients (35.3%) with a score ≥ 2. Nutritional status, evaluated using the GNRI with a cut-off of 81.6, classified 88 patients (86.3%) as High GNRI (≥ 81.6) and 14 (13.7%) as Low GNRI (< 81.6). The majority had advanced-stage disease, with 59 (57.8%) at Ann Arbor stage III-IV and 43 (42.2%) at stage I-II. According to the IPI, 58 patients (55.8%) were high or high-intermediate risk, including 40 (38.2%) high risk and 18 (17.6%) high-intermediate risk. Histologically, 29 patients (28.4%) were GCB type, 70 (68.6%) non-GCB type, and 3 (2.9%) of unknown origin.

**Table 1:** Baseline characteristics (*n* = 102)

Characteristics	*n* (%)
Age, years	
Median (IQR)	83.0 (81.0–85.0)
< 85	69 (67.6)
≥ 85	33 (32.4)
Sex	
Male	66 (64.7)
Female	36 (35.3)
Performance status	
ECOG 0–1	64 (62.7)
ECOG ≥ 2	38 (37.3)
CCI	
0–1	66 (64.7)
≥ 2	36 (35.3)
Serum albumin, g/dL	
Median (IQR)	3.6 (3.0–4.0)
eGFR, mL/min/1.73 m^2^	
≥ 60	72 (70.6)
< 60	30 (29.4)
GNRI	
High GNRI (≥ 81.6)	88 (86.3)
Low GNRI (< 81.6)	14 (13.7)
LDH, IU/L	
Normal	42 (41.2)
Abnormal	60 (58.8)
Extranodal site	
0–1	68 (66.7)
≥ 2	34 (33.3)
Ann Arbor stage	
I-II	43 (42.2)
III-IV	59 (57.8)
IPI	
Low	22 (21.6)
Low-intermediate	22 (21.6)
High-intermediate	18 (17.6)
High	40 (38.2)
Cell of origin	
GCB type	29 (28.4)
Non-GCB type	70 (68.6)
Unknown	3 (2.9)

*IQR* interquartile range, *ECOG* Eastern Cooperative Oncology Group, *CCI* Charlson comorbidity index, *eGFR* estimated glomerular filtration rate, *GNRI* geriatric nutritional risk index, *LDH* lactate dehydrogenase, *IPI* international prognostic index, *GCB* germinal center B-cell type

### Treatment delivery and outcomes

Following baseline assessment, we evaluated treatment delivery and clinical outcomes. Table 2 demonstrates treatment, delivery and outcomes. Among the 102 patients, the median number of R-CHOP cycles administered was 6 (IQR, 4–6), with a median total RDI of 51.6% (IQR, 49.3–64.7) for doxorubicin and 55.1% (IQR, 50.6–67.4) for cyclophosphamide, reflecting significant dose reductions. The overall median ARDI was 54.0% (IQR, 50.1–64.8). Treatment completion was achieved in 67 patients (65.7%), while 35 (34.3%) discontinued early, with intolerance being the most common reason (21 patients, 60.0%), followed by change of treatment modality (6, 17.1%), disease progression (5, 14.3%), and follow-up loss (3, 8.6%). Among the 93 evaluable patients, 76 (81.7%) achieved a complete response (CR), 3 (3.2%) a partial response (PR), and 14 (15.1%) experienced progressive disease (PD). Nine patients were not evaluable for response assessment due to follow-up loss (5 patients), pneumonia (2 patients), septic shock (1 patient), and respiratory failure (1 patient). Treatment-related toxicities of grade 3 or 4 occurred in 34 patients (33.3%), with non-hematologic toxicities (27, 26.5%) being more frequent than hematologic toxicities (23, 22.5%).

**Table 2:** Characteristics and outcomes of R-CHOP treatment in patients (*n* = 102)

Characteristics	*n* (%)
Cycle of R-CHOP	
Median (IQR)	6 (4–6)
Total RDI of doxorubicin, %	
Median (IQR)	51.6 (49.3–64.7)
Total RDI of cyclophosphamide, %	
Median (IQR)	55.1 (50.6–67.4)
Total ARDI, %	
Median (IQR)	54.0 (50.1–64.8)
Completion of R-CHOP	
Yes	67 (65.7)
No	35 (34.3)
Final response to R-CHOP *	
CR	76 (81.7)
PR	3 (3.2)
SD	0 (0)
PD	14 (15.1)
Reasons for early termination of R-CHOP	
Intolerance	21 (60.0)
Change of treatment modality (e.g. radiation)	6 (17.1)
Disease progression	5 (14.3)
Follow-up loss	3 (8.6)
Any toxicity, grade 3 or 4	
No	68 (66.7)
Yes	34 (33.3)
Hematologic toxicity, grade 3 or 4	
No	79 (77.5)
Yes	23 (22.5)
Non-hematologic toxicity, grade 3 or 4	
No	75 (73.5)
Yes	27 (26.5)

*R*-*CHOP* rituximab, cyclophosphamide, doxorubicin, vincristine, and prednisone, *IQR* interquartile range, *RDI* relative dose intensity, *ARDI* average relative dose intensity, *CR* complete response, *PR* partial response, *SD* stable disease, *PD* progressive disease

*Nine patients were not evaluable for response assessment

Among the 102 patients, 11 (10.8%) received consolidation radiotherapy. Of these, 7 patients (63.6%) received radiotherapy due to intolerance to R-CHOP, leading to early treatment termination, while 4 patients (36.4%) received planned consolidative radiotherapy after completing at least 4 cycles of R-CHOP. Radiotherapy was primarily administered for limited-stage (Ann Arbor I-II) disease (8/11, 72.7%) or for residual masses post-chemotherapy (3/11, 27.3%), with a median dose of 36.0 Gy (IQR 30.5–41.5 Gy). No grade 3–4 radiation-related toxicities were reported.

### Comparison between completed and incomplete treatment groups

The comparison between patients completing (*n* = 67) and those with incomplete R-CHOP cycles (*n* = 35) revealed significant differences in several characteristics, as shown in Table 3. Patients in the incomplete treatment group were significantly older (median age 84.0 vs. 82.0 years, *p* = 0.036) and had poorer performance status (ECOG PS ≥ 2: 54.3% vs. 28.4%, *p* = 0.018). The incidence of grade 3–4 non-hematologic toxicity was markedly higher in the incomplete group (48.6% vs. 14.9%, *p* = 0.001), with overall grade 3–4 toxicity also occurring more frequently (54.3% vs. 22.4%, *p* = 0.003), though hematologic toxicity showed no significant difference (*p* = 0.422). Notably, total ARDI and nutritional status (GNRI) were similar between groups, and no significant difference was observed in sex.

**Table 3:** Comparison of characteristics between patients completing and those with incomplete R-CHOP cycles

Variable	Completed cycles of *R*-CHOP (*n* = 67)	Incomplete cycles of *R*-CHOP (*n* = 35)	*p*-value
Age, years			0.036
Median (IQR)	82.0 (81.0–84.5)	84.0 (82.0–86.0)	
< 85	50 (74.6%)	19 (54.3%)	0.063
≥ 85	17 (25.4%)	16 (45.7%)	
Sex			0.419
Male	41 (61.2%)	25 (71.4%)	
Female	26 (38.8%)	10 (28.6%)	
GNRI			0.548
High GNRI (≥ 81.6)	59 (88.1%)	29 (82.9%)	
Low GNRI (< 81.6)	8 (11.9%)	6 (17.1%)	
Total ARDI, %			0.868
Median (IQR)	54.3 (50.1–67.1)	52.9 (50.4–63.5)	
ECOG PS			0.018
PS 0 ~ 1	48 (71.6%)	16 (45.7%)	
PS ≥ 2	19 (28.4%)	19 (54.3%)	
Any Toxicity, grade 3 or 4			0.003
No	52 (77.6%)	16 (45.7%)	
Yes	15 (22.4%)	19 (54.3%)	
Hematologic toxicity, grade 3 or 4			0.422
No	54 (80.6%)	25 (71.4%)	
Yes	13 (19.4%)	10 (28.6%)	
Non-hematologic toxicity, grade 3 or 4			0.001
No	57 (85.1%)	18 (51.4%)	
Yes	10 (14.9%)	17 (48.6%)	

*R*-*CHOP* rituximab, cyclophosphamide, doxorubicin, vincristine, and prednisone, *GNRI* geriatric nutritional risk index, *PS* performance status

Among patients experiencing non-hematologic grade 3–4 toxicities, early termination of R-CHOP chemotherapy was observed in multiple instances, predominantly attributable to declining performance status or severe infectious/inflammatory complications. Notably, treatment discontinuation after cycle 1 was associated with poor PS and follow-up loss, whereas in later cycles (C4-C5), causes included COVID-19 pneumonia, neutropenic fever, and complications such as cholecystitis, septic shock, and gastrointestinal bleeding. Several patients transitioned to involved-site radiation therapy or hospice care due to deteriorating clinical status related to infections or bleeding. These findings highlight the significant impact of infectious and treatment-related complications.

### Survival outcomes and prognostic factors

**Fig. 1:**
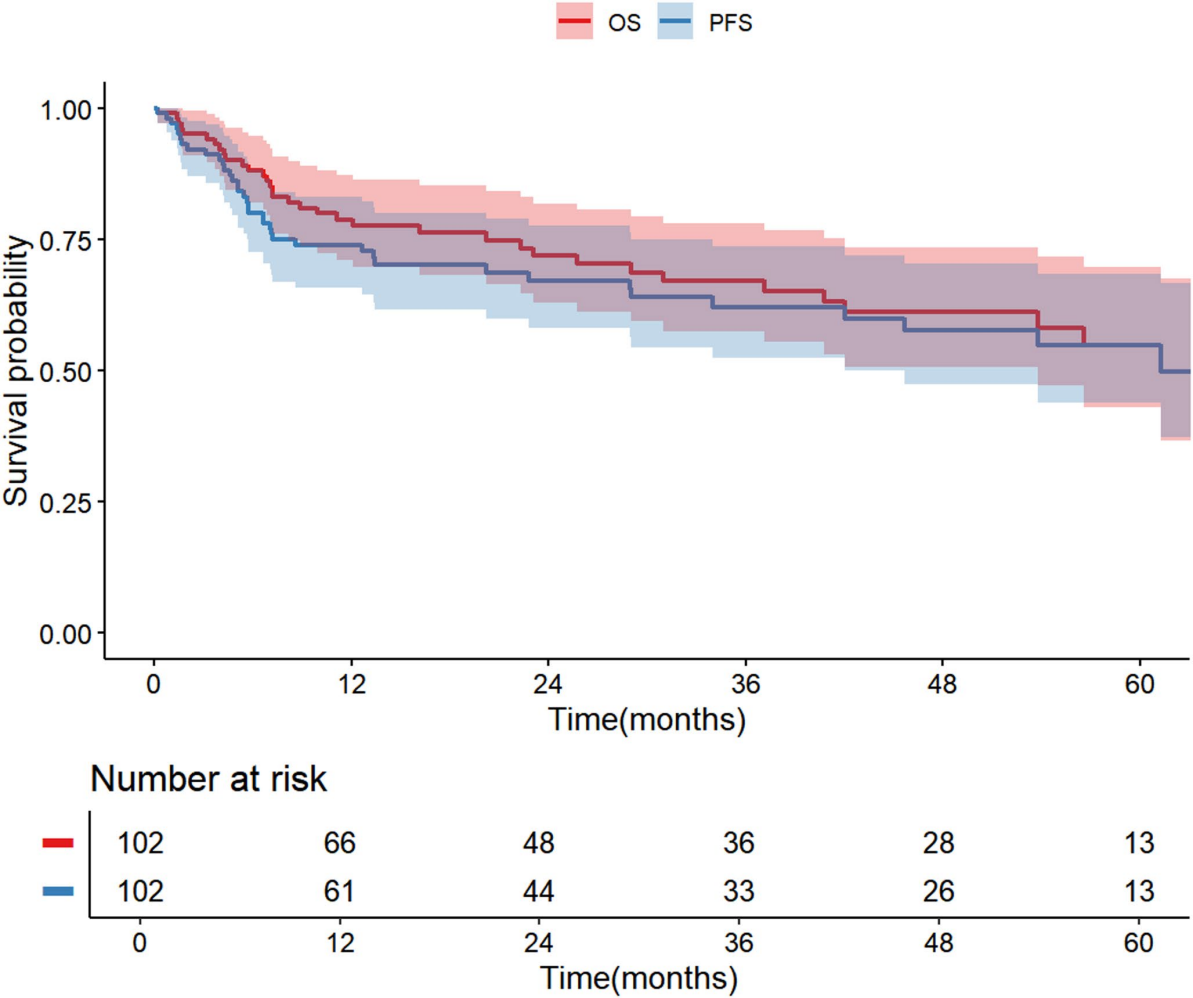
Kaplan–Meier Estimates of PFS and OS

The median follow-up duration, estimated using the reverse Kaplan-Meier method, was 39.1 months. Figure 1 shows Kaplan–Meier Estimates of PFS and OS up to 60 Months. At 2 years, the estimated PFS rate was 67.2% (95% CI, 58.1%–77.6%), and the OS rate was 71.8% (95% CI, 62.9%–81.8%). At 5 years, the estimated PFS rate was 54.8% (95% CI, 43.9%–68.4%), and the OS rate was 54.7% (95% CI, 43.0%–69.6%).

**Fig. 2:**
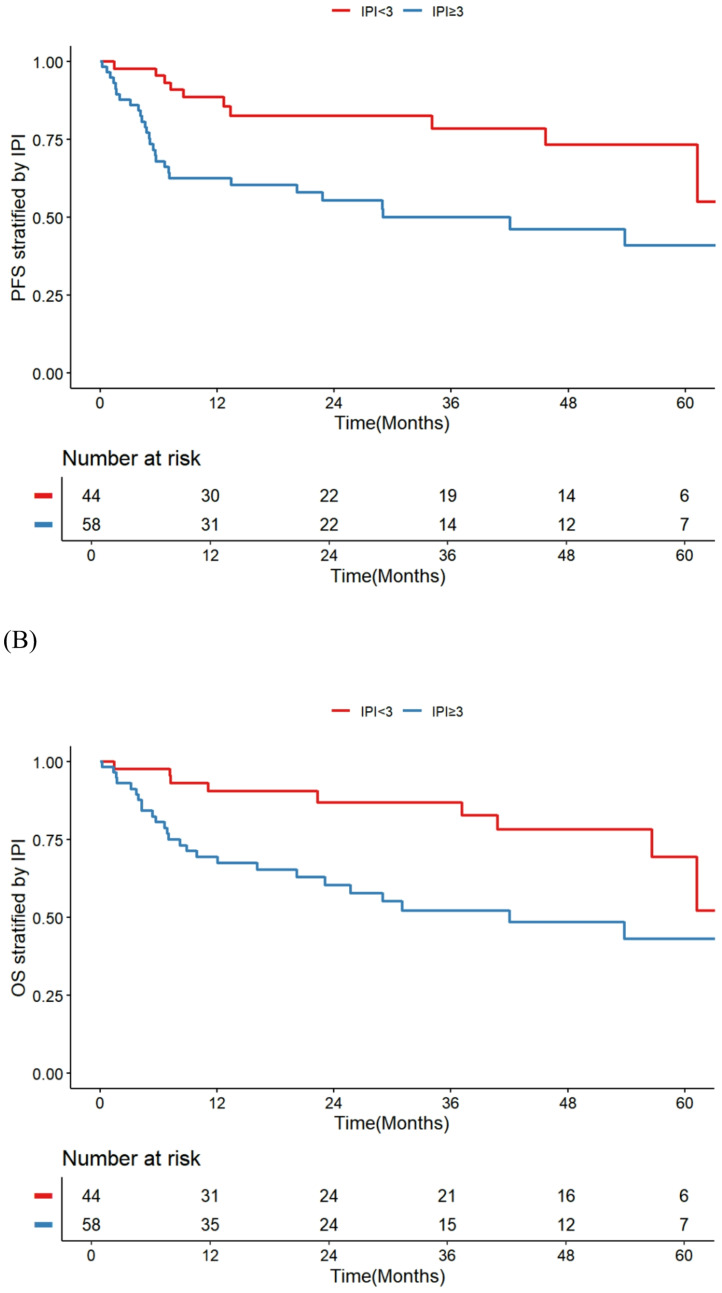
Kaplan–Meier Curves Stratified by IPI (A) PFS, (B) OS (A)

**Fig. 3:**
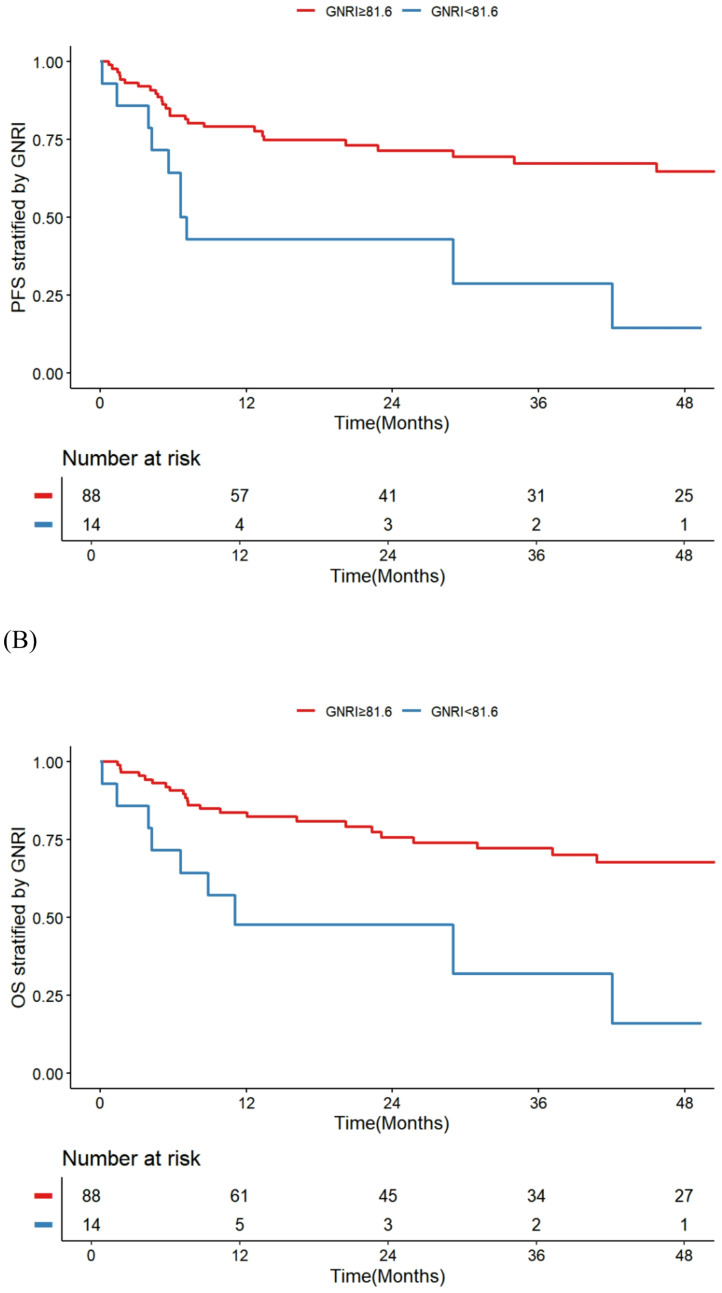
Kaplan–Meier Curves Stratified by GNRI (A) PFS, (B) OS (A)

Survival outcomes and prognostic factors were analyzed using univariate and multivariate Cox proportional hazards regression models (Table 4). In univariate analysis, IPI scores of ≥ 3 (HR 2.45, 95% CI 1.21–4.94, *p* = 0.012) and low GNRI (< 81.6; HR 3.43, 95% CI 1.64–7.16, *p* = 0.001) were significantly associated with inferior PFS, while male sex, CCI ≥ 2, non-GCB cell of origin, and total ARDI < 50% showed no significant associations. Similarly, for OS, IPI ≥ 3 (HR 2.43, 95% CI 1.17–5.06, *p* = 0.018) and low GNRI (HR 3.72, 95% CI 1.71–8.10, *p* < 0.001) emerged as significant predictors in univariate models. On multivariate analysis adjusting for relevant factors, low GNRI remained an independent predictor of both worse PFS (HR 2.84, 95% CI 1.34–6.02, *p* = 0.006) and OS (HR 3.09, 95% CI 1.40–6.82, *p* = 0.005), whereas IPI ≥ 3 independently predicted inferior PFS (HR 2.11, 95% CI 1.03–4.32, *p* = 0.042) but showed only borderline significance for OS (HR 2.09, 95% CI 0.99–4.41, *p* = 0.055). These findings underscore the prognostic value of nutritional status via GNRI in elderly DLBCL patients, independent of traditional risk factors. Figure 2 shows Kaplan-Meier survival curves for PFS and OS stratified by IPI groups, and Fig. 3 depicts curves stratified by GNRI groups.

**Table 4:** Univariate and multivariate analyses of survival in elderly DLBCL patients

	PFS						OS					
	Univariate			Multivariate			Univariate			Multivariate		
	HR	95% CI	*p*	HR	95% CI	*p*	HR	95% CI	*p*	HR	95% CI	*p*
Male sex	1.26	0.66–2.41	0.478	–	–	–	1.66	0.82–3.35	0.159	–	–	–
CCI ≥ 2	1.12	0.59–2.12	0.728	–	–	–	1.15	0.59–2.23	0.675	–	–	–
IPI ≥ 3	2.45	1.21–4.94	0.012	2.11	1.03–4.32	0.042	2.43	1.17–5.06	0.018	2.09	0.99–4.41	0.055
Cell of origin: non-GCB	0.92	0.45–1.86	0.813	–	–	–	0.82	0.40–1.69	0.595	–	–	–
Low GNRI (< 81.6)	3.43	1.64–7.16	0.001	2.84	1.34–6.02	0.006	3.72	1.71–8.10	< 0.001	3.09	1.40–6.82	0.005
Total ARDI < 50%	0.93	0.44–1.97	0.863	–	–	–	0.86	0.39–1.90	0.717	–	–	–

*PFS* progression-free survival, *OS* overall survival, *HR* hazard ratios; confidence interval, *CCI* Charlson comorbidity index, *GNRI* geriatric nutritional risk index, *GCB* germinal center B-cell type, *IPI* international prognostic index, *RCHOP* rituximab, cyclophosphamide, doxorubicin, vincristine, and prednisone, *ARDI* average relative dose intensity

The GNRI cut-off of 81.6, derived using maximally selected rank statistics, was subject to bootstrap validation (B = 500). Bootstrap-derived optimal cut-offs had a median of 84.39 (IQR 81.62–94.63 for OS; 81.62–96.78 for PFS), with 38–41% within ± 2 GNRI points and ~ 72% within ± 5 GNRI points of 81.6. At the fixed cut-off of 81.6, log-rank *p* < 0.05 occurred in 87% of resamples for both OS and PFS.

### Composite GNRI-IPI prognostic score

**Fig. 4:**
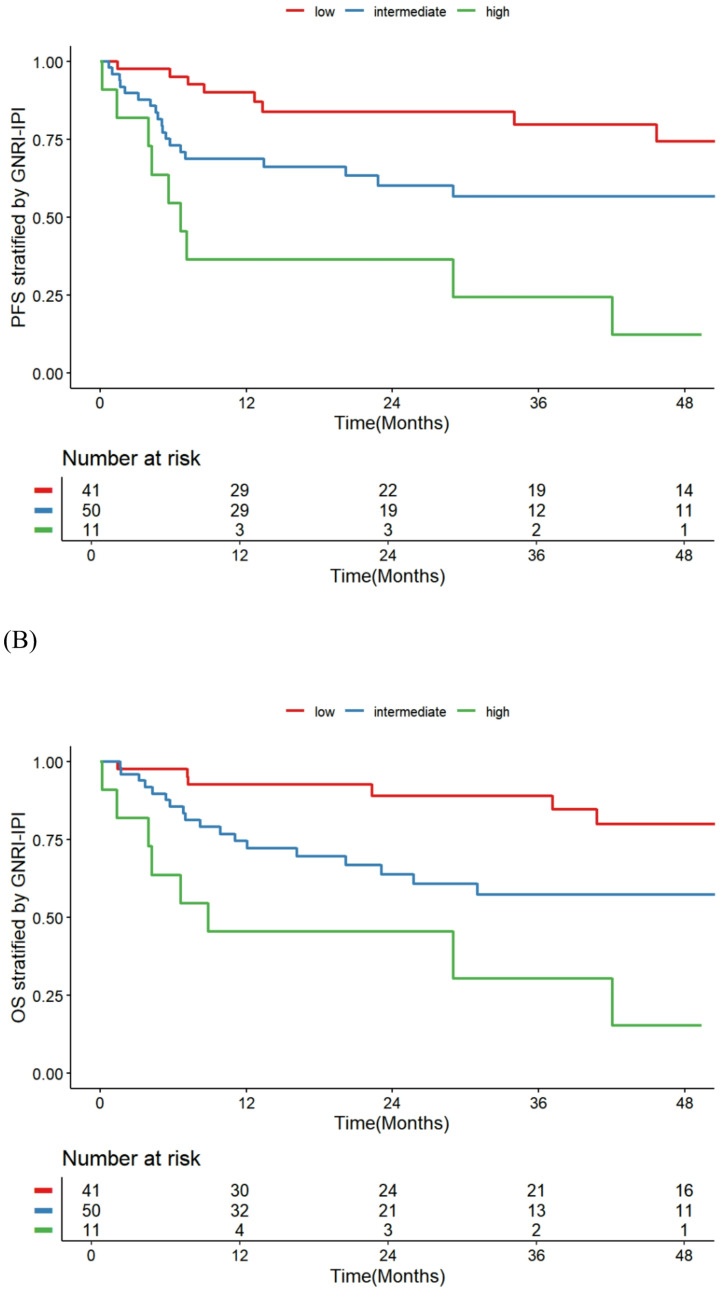
Kaplan–Meier Curves Stratified by the composite GNRI-IPI Score (A) PFS, (B) OS (A)

Patients were stratified into three risk groups based on GNRI and IPI scores as follows: Low-risk: GNRI ≥ 81.6 and IPI < 3 (*n* = 41, 40.2%) Intermediate-risk: Either GNRI < 81.6 or IPI ≥ 3 (*n* = 50, 49.0%) High-risk: GNRI < 81.6 and IPI ≥ 3 (*n* = 11, 10.8%). The composite GNRI-IPI score effectively stratified clinical outcomes across risk groups in elderly DLBCL patients (Table 5). For OS, the intermediate-risk group showed a non-significant increase in hazard compared to the low-risk group (HR 2.06, 95% CI 0.93–4.59, *p* = 0.077), while the high-risk group exhibited significantly worse OS (HR 6.44, 95% CI 2.45–16.93, *p* < 0.001). Similarly, PFS demonstrated a borderline trend toward inferiority in the intermediate-risk group (HR 2.01, 95% CI 0.94–4.33, *p* = 0.073) and a marked decline in the high-risk group (HR 6.00, 95% CI 2.42–14.92, *p* < 0.001) relative to low-risk patients. Kaplan-Meier survival curves showed clear separation among the three groups for both OS (log-rank *p* < 0.001) and PFS (log-rank *p* < 0.001) (Fig. 4). 4-year OS was 80.3% (95% CI, 66.3–96.6%) for the low-risk group, 57.2% (95% CI, 43.5–75.2%) for the intermediate-risk group, and 15.2% (95% CI, 2.7–8.5%) for the high-risk group, demonstrating a stepwise decrease in survival. While the GNRI-IPI score demonstrated slightly lower discrimination ability than the IPI alone, with Harrell’s C-index values of 0.683 vs. 0.734 for OS and 0.672 vs. 0.739 for PFS, the differences were modest and not considered clinically significant. At 24 months, calibration plots showed reasonable agreement between predicted and observed risks for both models. IPI demonstrated higher discrimination (AUC 0.737 [95% CI 0.629–0.844] vs. 0.676 [95% CI 0.566–0.787]) and a slightly lower Brier score (0.189 [95% CI 0.150–0.228] vs. 0.202 [95% CI 0.158–0.245]) (Supplementary Fig. 1). GNRI-IPI showed reasonable mid-range calibration but tended to overestimate risk in the high-risk tail, whereas IPI tracked the 45° line more closely at higher risk levels. We complemented the C-index with decision curve analysis at 24 months using time-specific binary outcomes and Cox-derived risks. Across clinically relevant thresholds (5–30%), the GNRI-IPI achieved higher net benefit than IPI, with ΔNB of − 0.011 at 5%, + 0.020 at 10%, + 0.120 at 20%, and + 0.052 at 30%; the average ΔNB over 5–30% was 0.0568, corresponding to roughly 5–6 additional true positives per 100 patients without increasing false-positive harm. These findings support the added clinical utility of the composite score, particularly at moderate-to-high thresholds (10–30%).

**Table 5:** Clinical outcomes by risk group based on the GNRI-IPI score

Outcome	Comparison	HR (95% CI)	*p*-value
OS	Intermediate vs. Low	HR 2.06 (0.93–4.59)	0.077
	High vs. Low	HR 6.44 (2.45–16.93)	< 0.001
PFS	Intermediate vs. Low	HR 2.01 (0.94–4.33)	0.073
	High vs. Low	HR 6.00 (2.42–14.92)	< 0.001
Early Chemotherapy Termination	High vs. Low	45.5% vs. 31.7%	0.482
Grade 3–4 Any Toxicity	High vs. Low	63.6% vs. 22.0%	0.023
Grade 3–4 Hematologic Toxicity	High vs. Low	36.4% vs. 12.2%	0.081
Grade 3–4 Non-Hematologic Tox.	High vs. Low	45.5% vs. 17.1%	0.100

*HR* hazard ratio, *CI* confidence interval, *OS* overall survival, *PFS* progression-free survival

Values are from Cox regression (for survival outcomes) and chi-square test (for toxicity and discontinuation)

Treatment-related outcomes showed similar early chemotherapy termination rates between high- and low-risk groups (45.5% vs. 31.7%, *p* = 0.482), but high-risk patients had significantly more grade 3–4 toxicities overall (63.6% vs. 22.0%, *p* = 0.023), with trends for higher hematologic (36.4% vs. 12.2%, *p* = 0.081) and non-hematologic toxicities (45.5% vs. 17.1%, *p* = 0.100). This underscores the GNRI-IPI score’s value in flagging patients at risk for worse survival and complications.

## Discussion

This study provides a comprehensive analysis of treatment outcomes and prognostic factors in patients aged ≥ 80 years with DLBCL treated with R-CHOP-based chemotherapy, addressing a critical gap in the management of very elderly patients. Our findings highlight the challenges of balancing treatment efficacy and toxicity in this population, with a median RDI of 54.0% reflecting significant dose reductions, consistent with prior studies reporting frequent dose attenuation due to comorbidities and frailty [10, 21, 22]. The 2-year OS of 71.8% and PFS of 67.2% in our cohort exceed those reported in earlier studies, such as the 2-year OS and PFS rates of 59% and 47% with R-miniCHOP in phase II trials [10, 21]. This improvement may reflect advances in supportive care, patient selection, or the inclusion of patients with better baseline characteristics, though direct comparisons are limited by our retrospective design.

A key finding is the prognostic significance of the GNRI, with a cut-off of 81.6 independently predicting worse PFS and OS (HR 2.84 and 3.09, respectively). The GNRI cut-off of 81.6 in our cohort was determined via maximally selected rank statistics (*p* = 0.022). This threshold is closely aligned with the value of 82 proposed by Bouillanne et al. [17] as the boundary for ‘major nutrition-related risk,’ a classification widely validated in elderly populations, including those with hematologic malignancies [23, 24]. Nutritional status, as assessed by GNRI, integrates serum albumin and body weight, capturing both malnutrition and frailty, which are prevalent in elderly patients [25, 26]. Our results align with studies demonstrating the prognostic value of nutritional indices in hematologic malignancies, suggesting that GNRI could complement the IPI in risk stratification for very elderly patients [23, 24, 27]. Our study confirms the prognostic value of the IPI in very elderly DLBCL patients. The IPI score ≥ 3 was an independent predictor of inferior PFS (HR 2.11, *p* = 0.042) and showed a trend toward worse OS (HR 2.64, *p* = 0.055) in multivariate analysis, reinforcing its established role in identifying high-risk patients, including those aged ≥ 80 years, as supported by prior studies [16, 28].

In this study, the composite GNRI-IPI score demonstrated potential in refining risk stratification for elderly patients with DLBCL undergoing R-CHOP therapy. By categorizing patients into low, intermediate, and high-risk groups, the score effectively delineated survival outcomes, with Kaplan-Meier analyses revealing significant separations in OS and PFS, alongside a stepwise decline in 4-year OS from 80.3% in the low-risk group to 15.2% in the high-risk group. Cox regression confirmed GNRI-IPI as an independent predictor of OS, underscoring the additive value of incorporating nutritional status (via GNRI) to traditional IPI factors, which may better capture frailty and treatment tolerance in geriatric populations. Although the GNRI-IPI’s C-index (0.683 for OS, 0.672 for PFS) was modestly lower than that of IPI alone (0.734 and 0.739, respectively), suggesting that GNRI-IPI may not surpass established models in overall discrimination due to overlapping prognostic elements or sample size limitations. Furthermore, the association of high-risk GNRI-IPI with elevated rates of grade 3–4 toxicities highlight its utility in anticipating adverse events and guiding supportive care strategies, though the trend toward non-hematologic toxicity requires further validation in larger cohorts. Overall, these findings advocate for the integration of nutritional metrics in lymphoma prognostication, potentially informing personalized therapeutic approaches, but emphasize the need for prospective studies to optimize and validate the GNRI-IPI score against existing tools.

The higher incidence of grade 3–4 non-hematologic toxicities in the incomplete treatment group (48.6% vs. 14.9%) underscores the profound impact of infectious and treatment-related complications on treatment adherence. These findings highlight the vulnerability of very elderly DLBCL patients to such toxicities, particularly infections and decline in performance status, which frequently lead to treatment interruption or escalation to palliative care. In this context, vigorous primary prophylaxis against infections should be recommended for octogenarians receiving anthracycline-based regimens. Prophylactic strategies, including long-acting granulocyte colony-stimulating factor (G-CSF), trimethoprim–sulfamethoxazole, and acyclovir, have been advocated to reduce febrile neutropenia and opportunistic infections in high-risk lymphoma patients [29]. Cardiotoxicity, alongside infectious complications, is a major concern in octogenarians with DLBCL receiving anthracycline-based regimens such as R-CHOP, with a planned cumulative doxorubicin dose of approximately 300 mg/m². Integration of cardio-oncology services is crucial, including baseline and serial echocardiography with left ventricular ejection fraction and global longitudinal strain monitoring, and early initiation of cardioprotective agents, such as angiotensin-converting enzyme inhibitors, angiotensin receptor blockers, and beta-blockers, in at-risk patients [30, 31]. Incorporating such approaches may help to minimize treatment-related morbidity and support treatment completion in this vulnerable population. This data further emphasizes the necessity of implementing early geriatric assessments and tailored supportive care interventions to optimize treatment tolerance and improve outcomes in this high-risk group. High risk of central nervous system (CNS) involvement warrants enhanced surveillance in DLBCL, a rare but devastating complication. Strategies include baseline CNS imaging and cerebrospinal fluid cytology for high-risk features, with prophylactic intrathecal methotrexate or systemic high-dose methotrexate recommended by NCCN guidelines [11]. However, their feasibility and tolerability are often limited in octogenarians due to frailty and toxicities, such as neurotoxicity or renal impairment. Consistent with reports by Derman et al., comprehensive geriatric assessment–guided multidisciplinary care has been shown to reduce non-relapse mortality by enabling early identification and proactive management of geriatric impairments, including infection risks and functional decline [32].

The stromal gene signature in large B-cell lymphoma, classified as GCB or activated B-cell–like (ABC), has long been established as an independent determinant of prognosis [33]. Our cohort had a predominance of the non–GCB subtype (68.6%), and we found that cell of origin (GCB vs. non-GCB) lacked prognostic significance. This may reflect the limited sample size or the overriding impact of age-related and disease-specific factors in the very elderly. Alternatively, IHC classifiers, unlike gene expression profiling, may not reliably stratify outcomes [34]. The POLARIX trial demonstrates that polatuzumab vedotin plus R-CHP (Pola-R-CHP) improves progression-free survival in ABC-DLBCL compared to R-CHOP, with reduced peripheral neuropathy [35]. However, data in patients ≥ 80 years are limited due to exclusion from pivotal trials. The GNRI-IPI score, integrating nutritional frailty, could be particularly valuable in identifying very elderly ABC-DLBCL patients suitable for Pola-R-CHP versus R-CHOP or R-miniCHOP, as low GNRI may predict higher toxicity with intensified regimens. Prospective studies evaluating GNRI-IPI in this context are needed.

Notably, approximately 42% of our patients had early-stage (Ann Arbor I-II) disease but received a median of 6 cycles of R-CHOP (full or attenuated), consistent with historical practices but potentially exceeding current international guidelines (e.g., NCCN and ESMO) for limited-stage disease, which recommend 3–4 cycles followed by involved-site radiotherapy in select younger patients with low-risk features [36, 37]. However, these trials primarily enrolled patients aged 18–60 years, with very limited inclusion of those ≥ 80 years, limiting direct applicability to our very elderly cohort. In the absence of randomized data comparing abbreviated versus full-course attenuated regimens in octogenarians with limited-stage DLBCL, LYSA studies support 6 cycles of R-miniCHOP as the standard [10], emphasizing the need for future prospective evaluations in this population to optimize de-escalation strategies while minimizing toxicity.

Our study has several limitations. Its retrospective, single-center design and relatively small sample size (*n* = 102) may introduce selection bias and limit generalizability, potentially reducing statistical power to detect associations for certain variables. However, for model stability, multivariable Cox analyses were restricted to two prespecified predictors (GNRI and IPI). Among 102 patients, we observed 38 deaths and 41 PFS events, yielding events-per-variable of 19.0 and 20.5, respectively (both ≥ 10), supporting the adequacy of the sample size and model reliability. The GNRI cut-off of 81.6 may be subject to overfitting despite internal bootstrap validation, with variability in optimal cut-offs indicating a need for external validation in larger cohorts. These 24-month analyses confirm IPI’s modest edge in discrimination and calibration, while GNRI-IPI provides complementary value through nutritional frailty assessment. External validation with NRI, IDI, and DCA in larger cohorts is needed to address derivation bias.

We did not perform comprehensive geriatric assessment (CGA) for frailty or cognition beyond ECOG PS. Future studies integrating CGA could refine GNRI-IPI further. The Elderly Prognostic Index (EPI), a validated tool integrating simplified geriatric assessment, including Activities of Daily Living (ADL) and Instrumental Activities of Daily Living (IADL), with traditional prognostic factors, enhances overall survival prediction in DLBCL patients aged ≥ 70 years treated with R-CHOP [15]. However, we could not compare EPI with GNRI-IPI due to the absence of ADL and IADL data in our retrospective cohort. The study period (2005–2024) spans nearly two decades, with evolving supportive care practices. Wider adoption of primary G-CSF prophylaxis, improved infection prevention strategies, and advances in supportive care may have potentially contributed to improved treatment tolerance and survival. These temporal changes could have influenced outcomes in our cohort; thus, results should be interpreted with caution. Despite these limitations, this study proposes a preliminary GNRI-IPI score that integrates nutritional status (GNRI) and disease-specific risk (IPI), offering a simplified prognostic index for very elderly DLBCL patients. Future studies should validate the GNRI-IPI score in larger, multicenter cohorts, integrate additional geriatric assessments (e.g., frailty, cognitive function), or employ machine learning to optimize risk stratification. Interventional trials targeting nutritional support or toxicity mitigation in high-risk GNRI-IPI groups are warranted.

In conclusion, our study underscores the prognostic importance of nutritional status (GNRI) and IPI ≥ 3 in very elderly DLBCL patients treated with R-CHOP. The composite GNRI-IPI score enhances risk stratification, predicting not only survival but also treatment tolerance and toxicity, offering a practical tool to guide personalized treatment strategies in this vulnerable population. Larger, prospective studies are needed to validate these results and explore innovative therapeutic approaches.

## Supplementary Information

Below is the link to the electronic supplementary material.

ESM 1  (DOCX 136 KB) 

## Data Availability

The datasets generated and/or analyzed during the current study are not publicly available due to patient confidentiality and institutional data protection policies. De-identified data may be made available from the corresponding author upon reasonable request and with approval from the Institutional Review Board of the institution.
