# Supplementary material for: Prognostic impact of GNRI-IPI score in octogenarians with diffuse large B-Cell lymphoma treated with R-CHOP
Source: Ann Hematol. 2025 Oct 14;104(11):5867–79. doi: 10.1007/s00277-025-06670-x (PMC12672595; doi:10.1007/s00277-025-06670-x)

**ORIGINAL ARTICLE**

**Prognostic Impact of GNRI-IPI Score in Octogenarians with Diffuse Large B-Cell Lymphoma Treated with R-CHOP**

Eun-Jeong Jeong^1^, Woochan Park^1^, Jeongmin Seo^1^, Minsu Kang^1^, Eun Hee Jung^1^, Sang-A Kim^1^, Koung Jin Suh^1^, Ji-Won Kim^1^, Se Hyun Kim^1^, Jeong-Ok Lee^1^, Jin Won Kim^1^, Yu Jung Kim^1^, Keun-Wook Lee^1^, Jee Hyun Kim^1^, Jong Seok Lee^1^, Soo-Mee Bang^1^, Ji Yun Lee^1^**†**

^1^Department of Internal Medicine, Seoul National University Bundang Hospital, Seongnam, Republic of Korea

**Running title:** R-CHOP Outcomes in Elderly DLBCL Patients

**† Address for correspondence:** Ji Yun Lee, MD, PhD

Department of Internal Medicine, Seoul National University Bundang Hospital, Gumi-ro 173 Beon-gil, Bundang-gu, Seongnam-Si, Gyeonggi-di 13620, Korea

Tel: +82-31-787-7443; Fax: +82-31-787-4098

E-mail: maimatin83@snubh.org

**Supplementary Figure 1.** **Calibration of 24-month Overall Survival Risk: IPI vs GNRI-IPI (AUC and Brier shown).**


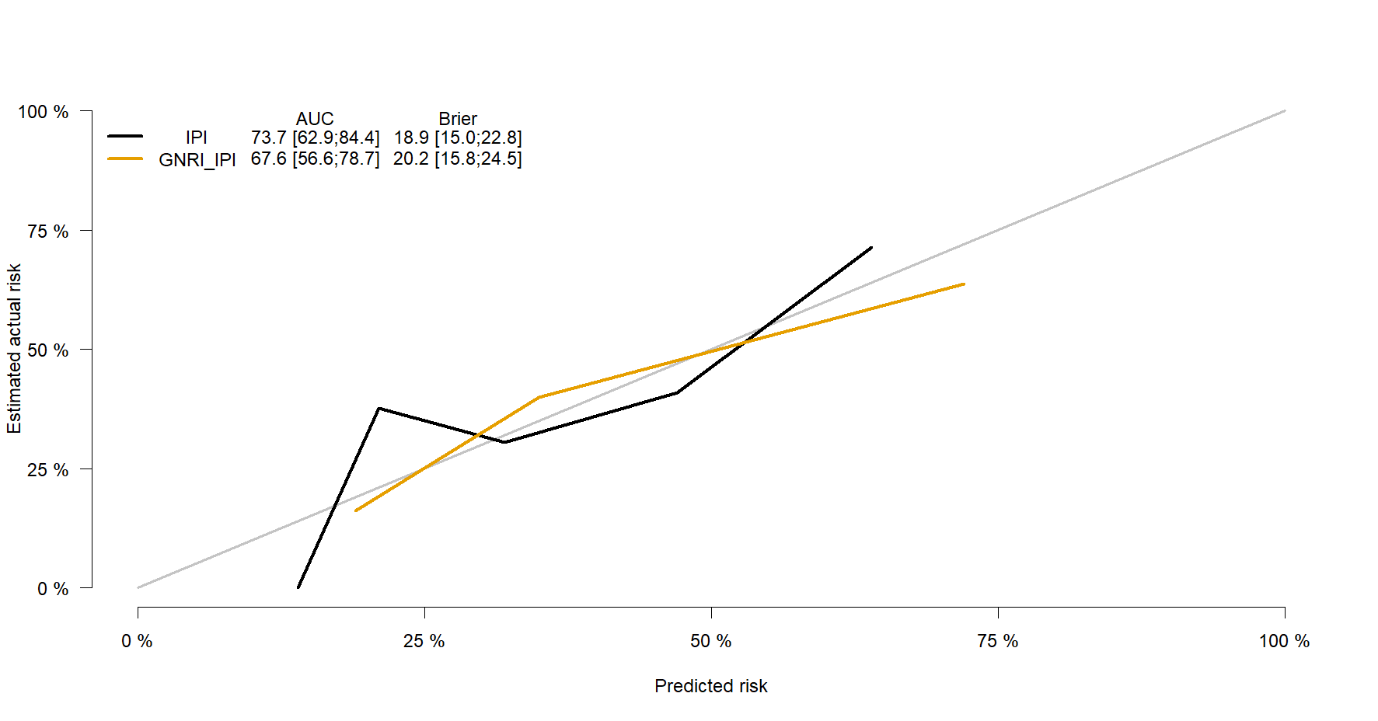

Supplement: Supplementary file 1 — (DOCX 136 KB) [file 277_2025_6670_MOESM1_ESM.docx]
